# Comparison of Antibacterial Effect of Cationic Peptide LL‐37 and Cefalexin on Clinical *Staphylococcus aureus‐*induced Infection after Femur Fracture Fixation

**DOI:** 10.1111/os.12754

**Published:** 2020-07-28

**Authors:** Cheng‐yuan Yan, Yu‐zhou Liu, Zhong‐hua Xu, Hao‐yu Yang, Jin Li

**Affiliations:** ^1^ Department of Orthopaedics Jintan Hospital Affiliated to Jiangsu University Changzhou China; ^2^ Department of Orthopaedics Wuxi 9th People's Hospital affiliated to Soochow University Wuxi China; ^3^ Department of Orthopaedic Surgery The Second Affiliated Hospital of Jiaxing University Jiaxing China

**Keywords:** Antimicrobial activity, Biofilm, Femoral fracture fixation, Rabbit model, *Staphylococcus aureus*

## Abstract

**Objective:**

Antimicrobial peptides are widely present in nature, with many of the antimicrobial peptides having antimicrobial activity against Gram‐positive and Gram‐negative bacteria, fungi, parasites, and even coated viruses. Internal fixation of fractures is a reliable technique. However, the fracture is difficult to heal and internal fixation is not easy to maintain after infection. This study aims to verify the antibacterial effect of cationic peptide LL‐37 on *Staphylococcus aureus*, explore the anti‐biofilm effects of LL‐37, and compare the effects of the cationic peptide LL‐37 and Cefalexin in treatment of postoperative infection of femoral fracture *in vivo*.

**Methods:**

The *Staphylococcus aureus* was clinically isolated from one patient with clinical infection after the fracture fixation at Wuxi 9th People's Hospital. The cationic peptide LL‐37 was synthesized by Shanghai Apeptide Co. Ltd. To compare the effects of the cationic peptide LL‐37 and Cefalexin in the treatment of postoperative infection of femoral fracture *in vivo*, 63 rabbits with internal fixation of femoral fractures were inoculated intravenously with clinically isolated pathogenic bacteria suspensions. Rabbits in the treatment groups were treated with peptide LL‐37 and Cefalexin after surgery. Rabbits in the control groups were treated with physiological saline after surgery. The biofilms on internal fixtures were harvested from euthanized rabbits 1 h, 12 h, 1 day, 2 days, and 7 days after injection of LL‐37, Cefalexin, or saline and calculated by colony count. The biofilms from treatment and control groups at 7 days were analyzed by fluorescence microscopy. Blood samples were collected at 1 h, 12 h, 1 day, 2 days, and 7 days following peptide LL‐37 and Cefalexin injection.

**Results:**

The results were compared statistically using Student's *t*‐test or two‐way analysis of variance (ANOVA). Cationic peptide LL‐37 showed significant inhibitory effects on clinically isolated *Staphylococcus aureus* (*P* < 0.05) compared with Cefalexin and control group at 1 day (*P* = 0.021), 2 days (*P* = 0.019), and 7 days (*P* = 0.025). Fluorescent images of the biofilm reveal that the numbers of cells on biofilms are far less than those in the Cefalexin and control groups at 7 days. The levels of Interleukin‐6 (IL‐6), tumor necrosis factor‐α (TNF‐α) and C‐reactive protein (CRP) reached a maximum at 2 days following the operation. After the injection of LL‐37, there was an increase in the serum IL‐6, TNF‐α, and CRP contents in rabbits in both groups, however from 1 day postoperative the level of IL‐6 (*P* = 0.034), TNF‐α (*P* = 0.043), and CRP (*P* = 0.039) decreased significantly compared to the Cefalexin and control group. At 7 days postoperative, the level of IL‐6 (*P* = 0.029), TNF‐α (*P* = 0.033), and CRP (*P* = 0.027) had reverted to normal levels in LL‐37 groups.

**Conclusions:**

Cationic peptide LL‐37 may be a promising agent to control internal femoral fracture fixation infection *in vivo*.

## Introduction

Fracture is a disruption or break in the continuity of the structure of a bone. Fracture occurs mainly in the elderly and children. Most patients can restore the original function of bone with timely and appropriate treatment; however, some patients will have potentially different outcomes. The application of internal fixation technique for fractures in clinical settings is very common, and although advanced equipment and necessary aseptic conditions have been developed, postoperative infection of the fractures undergoing internal fixation is still an inevitable complication^1^. Infection is one of the major factors that affects fracture healing^1^, ^2^, often leading to acute fracture hematoma infection or chronic osteomyelitis.

Internal fixation of fractures is a reliable technique^3^. However, once the infection occurs, the fracture is difficult to heal and internal fixation is not easy to maintain, thus necessitating a strict aseptic environment for the surgery. Infection of the implanted internal fixator is mainly caused by adherent bacteria^4^, suggesting that elimination of bacterial biofilm is important for treatment of infections.

In recent years, while the use of antibiotics in large doses has reduced infection rates, it has also promoted development of drug resistance of pathogenic microorganisms^5^. For example, the dosages of the penicillin now used in the treatment of bone infection are several dozens of times more than those used when penicillin was first discovered^6^. This is a matter of serious concern as there is emergence of superbugs that do not respond to any antibiotics^7^. At the same time, an increase in the use of antitumor and broad‐spectrum antibacterial agents, along with improved survival of AIDS and immunocompromized patients, increases the chances of more serious infections in transplants. The phenomenon of bacterial resistance is becoming common, it is closely related to the abuse of antibiotics; however, it is also closely related to the biofilm‐formed bacteria. Biofilms are antibiotic resistant colonization of bacteria that attach to surfaces and form a slime‐like barrier that acts as a formidable defense mechanism, protecting the bacteria from eradication. *Staphylococcus aureus* is the important pathogen of nosocomial infection and the leading cause of biofilm infection. *Staphylococcus aureus* easily adheres to the surface of orthopaedic implants, therefore, seeking an effective drug to resist biofilm in the treatment of orthopaedic infection is really urgent.

In finding an effective weapon against these infectious diseases, the scientists have focused on the exploration of antibacterial peptides. Antibacterial peptides are one kind of natural protein, which can kill bacteria, viruses and fungi, and researchers have been trying to use antibacterial peptides to replace traditional antibiotics. Natural antibacterial peptides usually consist of 20 kinds of amino acids and the antibacterial peptides can exert a specific function through the adjustment of the order of amino acids. Antimicrobial peptides are widely present in nature, with many of the antimicrobial peptides having antimicrobial activity against Gram‐positive and Gram‐negative bacteria, fungi, parasites, and even coated viruses^8^. They have an important regulatory role in the host innate immune and adaptive responses and have a unique antibacterial mechanism that is different from traditional antibiotics, making them ideal candidates for developing new antibiotic drugs. In order to ensure the stabilization in the body's internal environment, biological immune cells have developed a variety of immune mechanisms; among them, the antibacterial peptide, as the main material secreted by the host cell, have played an important function as the “safeguard” in the process of invasion against pathogens. The mechanism of antimicrobial peptides in the antimicrobial therapy is unlike antibiotics; the antimicrobial peptides could mainly destroy the integrity of the cell membrane of pathogenic bacterium to bring the cell death of bacteria. Cationic peptide LL‐37 could be produced by bone marrow and testis, inflamed skin keratinocytes, lung epithelia, and squamous epithelia of human mouth, tongue, esophagus, cervix, and vagina and is usually present in neutrophil granules. Studies had shown that both purified and chemically synthesized cationic LL‐37 peptides could exhibit potent and comparable antimicrobial activities *in vitro* ^9^. Meanwhile, there have been few studies on the use of cationic peptide LL‐37 in the control of internal femoral fracture fixation infection *in vivo*. Therefore, to explore the cationic peptide LL‐37 in the control of internal femoral fracture fixation infection *in vivo* seems really meaningful in the orthopaedic treatment of clinical infection.

We aim to: (i) verify the antibacterial effect of cationic peptide LL‐37 in clinically isolated *Staphylococcus aureus*; and (ii) compare the effects of the cationic peptide LL‐37 and Cefalexin in the treatment of postoperative infection of femoral fracture *in vivo* and (iii) invest the improvement of inflammatory factor after the injection of cationic peptide LL‐37, Cefalexin, and physiological saline. In order to verify these effects we isolated one *S. aureus* strain from one patient with clinical infection after the fracture fixation, analyzed its biofilm formed by clinically isolated *S. aureus* in infected femoral fracture of rabbit model, and measured the serum concentrations of IL‐6, TNF‐α, and CRP in rabbits with *S. aureus* infections.

## Materials and Methods

### *Bacterial Isolation and Culture*

The project was approved by the Ethical Committee of Wuxi 9th People's Hospital affiliated to Soochow University. One patient with clinical infection after fracture fixation at Wuxi 9th People's Hospital was selected to extract the clinical pathogenic bacteria. Bacterial specimens were obtained after washing the surface of the wound vigorously with saline, followed by debridement of superficial exudates, and were cultured under microaerophilic conditions for 7 days.

### *Identification of Bacteria Using the VITEK 2 Fluorescent System (ID‐GNB Card)*

The isolated stock culture strains were subcultured onto MacConkey agar plates to check their purity. The turbidity of the bacterial suspensions was adjusted with a densitometer to a McFarland 0.5 standard in 0.45% sterile sodium chloride solution. The suspensions were then transferred to VITEK 2 ID‐GNB cards and AST‐No. 12 cards and manually loaded into the VITEK 2 system. The data was analyzed by the VITEK 2 system and the results were reported automatically.

### *Peptide*

Cationic peptide LL‐37 (LLGDFFRKSKEKIGKEFKRIVQRIKDFLRNLVPRTES) was synthesized by Shanghai Apeptide Co. Ltd. (Shanghai, China). LL‐37 was purified by high‐performance liquid chromatography and the identity was verified by SDS‐PAGE. The purity of LL‐37 (>95%) and mass were confirmed by electrospray ionization mass spectrometry.

### *Animals*

A total of 63 New Zealand white rabbits (weighing 2.0 to 2.5 kg) were used in this study. Twenty‐one rabbits in the control groups were treated with physiological saline after surgery, and 21 rabbits in the treatment groups were treated with LL‐37 and Cefalexin after surgery.

### *Model Validation*

Rabbits were anesthetized *via* inhalation of isoflurane (2%). The surgical procedure was as described in the previous work with few modifications^10^. The skin was incised at the proximal end of the specimen so as to facilitate loading directly longitudinally over the lateral upper forelimb. Dissection was continued up to the fascia of the biceps and brachialis, which were then retracted to reveal the midshaft humerus. A 3.5 mm diameter twist drill was used to ream the medullary canal both antegrade and retrograde from the osteotomy site. Using the greater tuberosity starting hole, a rod was placed across the fracture site, down the medullary canal and into the distal fragment. Excess rod length was removed at the greater tuberosity with a hand‐held pin cutter.

An inoculum of clinically isolated *S. aureus* (1 × 10^5^ cfu/mL) in 2 mL of normal saline was pipetted into the femur space containing the cut end of the implant^11^. The surgical site was closed with Dexon 5–0 sutures.

### *Detection of Biofilms*

To evaluate biofilm formation on the femoral fracture internal fixation in our rabbit model, the biofilms on internal fixtures were harvested from euthanized rabbits 1 h, 12 h, 1 day, 2 days, and 7 days after injection of LL‐37, Cefalexin, or saline. Each sample was washed in 10 mL of phosphate‐buffered saline (PBS) and then 0.1 mL of the suspension were inoculated and counted for the colonies on Columbia Blood Agar Base.

After 7 days, internal fixtures were extracted and fixed with 2.5% glutaraldehyde at 4°C for 1 h. After fixation, the internal fixtures were stained using BacLight Live/Dead staining kit (Invitrogen, Carlsbad, CA) and observed under a Nikon 80i fluorescence microscope at an excitation wavelength of 488 nm.

### *Determination of Serum Bactericidal Titer of Rabbits*

Blood samples were collected 1 h, 12 h, 1 day, 2 days, and 7 days after injection of peptide LL‐37, Cefalexin, or application of saline. The samples were centrifuged at 4000 r/min for 3 min and stored at −70°C. The serum interleukin‐6 (IL‐6), tumor necrosis factor‐α (TNF‐α), and C‐reactive protein (CRP) levels were analyzed using ELISA and expressed as pg./mg protein^12^.

### *Statistics Analysis*

All tests were repeated three times for consistency of results. SPSS 14.0 software for Windows was used for data analysis. The results were compared statistically using Student's *t*‐test or two‐way analysis of variance (ANOVA). Values were considered statistically significant at *P* < 0.05.

## Results

### *Biofilm Formation on the Femur*

**Fig. 1:**
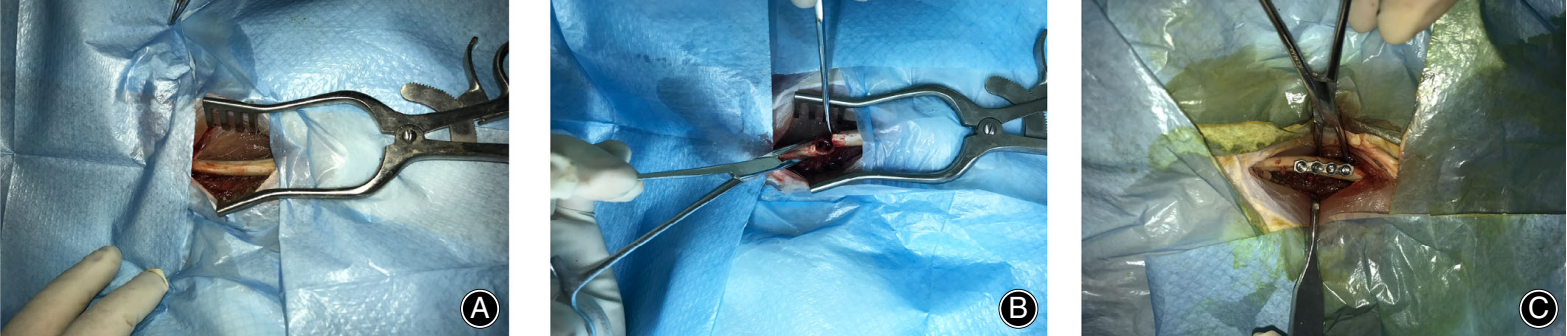
The surgery of femoral fracturefixation in rabbit model. (A) the exposure of the femur; (B) artificial femoral fractures; (C) femoral fracture fixation.

**Fig. 2:**
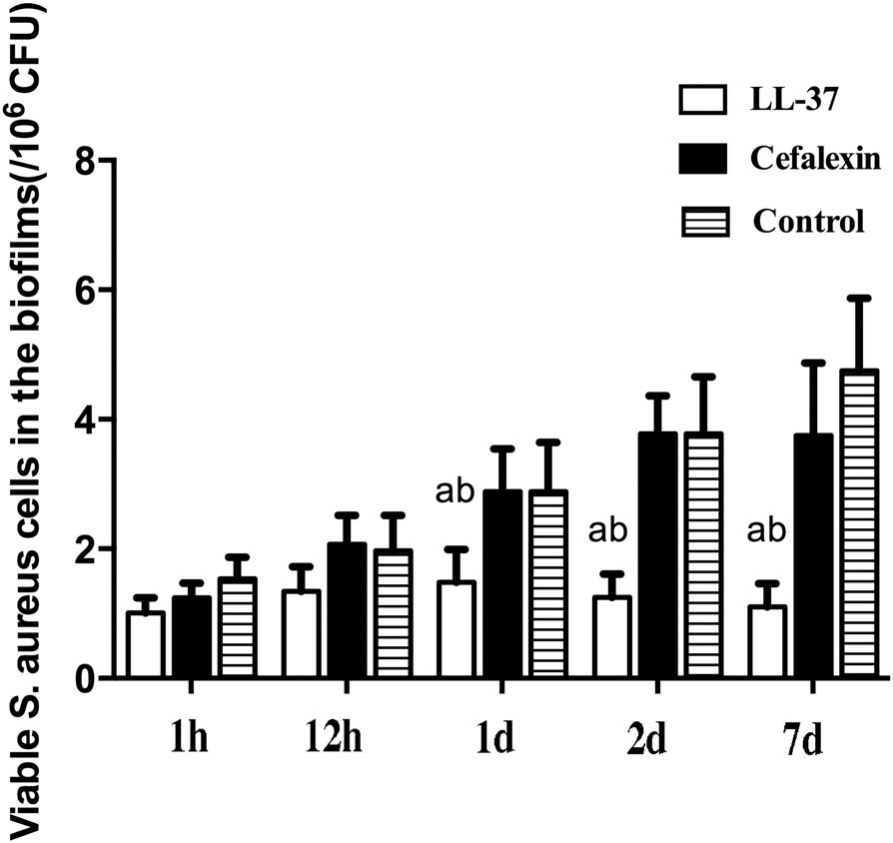
The colony count of biofilms in PBS‐control, Cefalexin rabbits and LL‐37‐treated rabbits after 1 h, 12 h, 1 day, 2 day, 7 day. (a) compared with PBS‐control group, *P* < 0.05; (b) compared with Cefalexin group, *P* < 0.05).

**Fig. 3:**
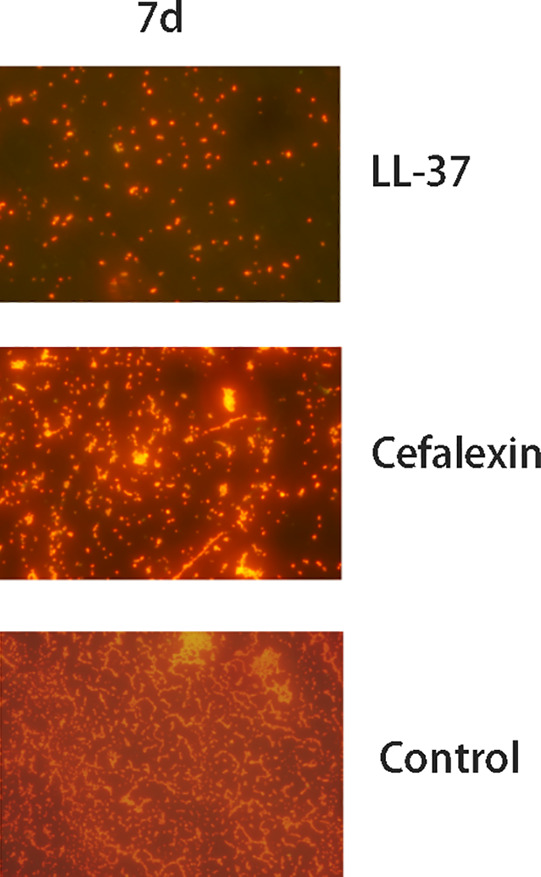
Fluorescent images of the biofilm of clinically isolated *S. aureus* in PBS‐control, Cefalexin rabbits and LL‐37‐treated rabbits after 7 day (Stained with BacLight Live/Dead staining kit). LL‐37‐treated groups showed less bacteria on the biofilm compared with PBS‐control and Cefalexin groups.

The surgical procedures were shown in Fig. 1. The pathogenic strain isolated from the patient was identified as *S. aureus* by using VITEK 2 fluorescent system. At 1 day following injection with peptide LL‐37, the colony count of the biofilm began to show the significant changes as compared to the Cefalexin and saline groups (*P* = 0.021; Fig. 2). Then the colony count of the biofilm decreased significantly compared to the Cefalexin and control groups at 2 days (*P* = 0.019; Fig. 2) and 7 days (*P* = 0.025; Fig. 2). Fluorescent images of the biofilm reveal that the numbers of cells on biofilms are far less than those in the Cefalexin and control groups at 7 days (Fig. 3).

### *Serum IL‐6, TNF‐α, and CRP Level of Inflammatory Cytokines in Different Groups*

**Fig. 4:**
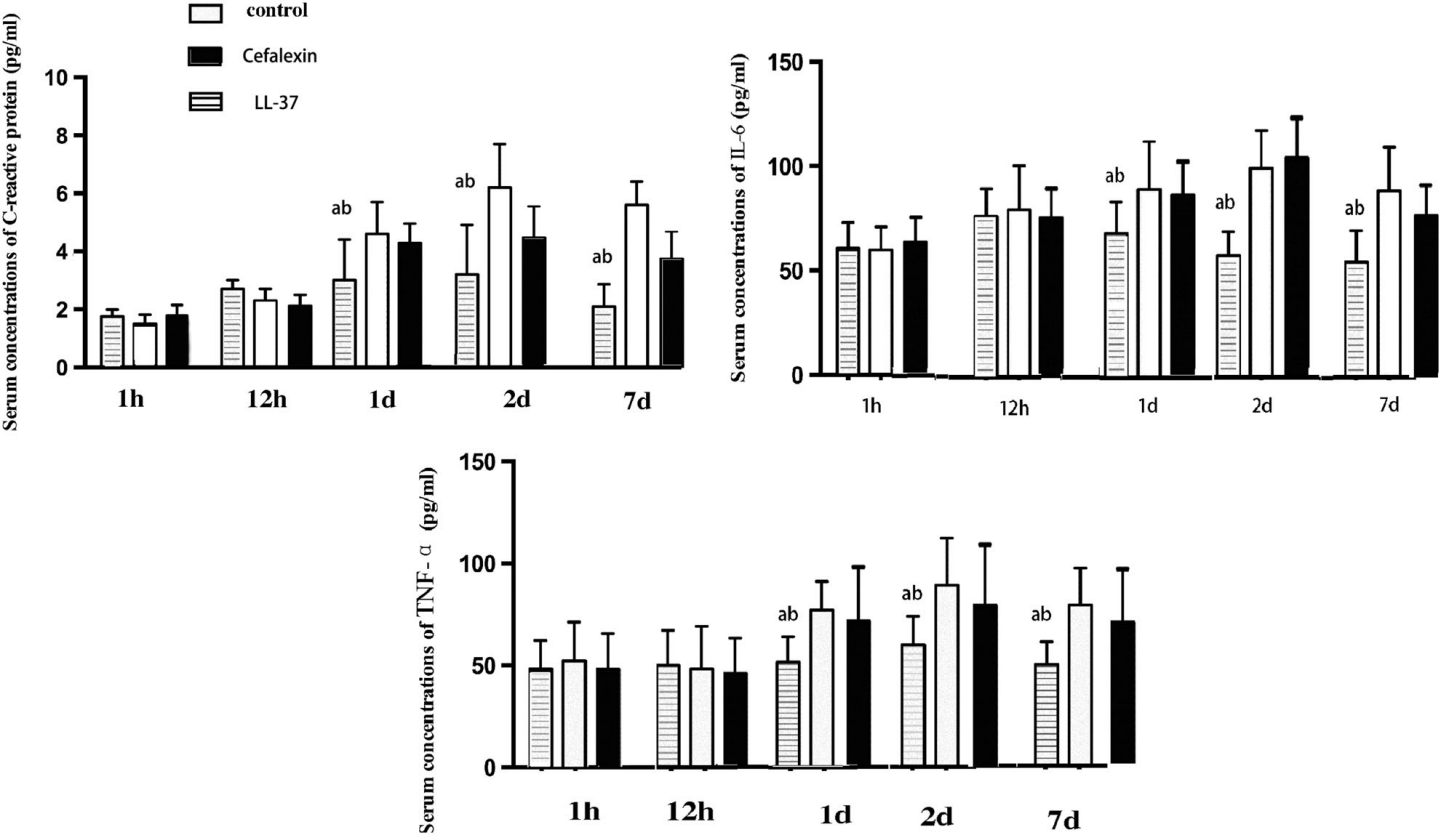
Serum concentrations of C‐reactive protein (CRP), IL‐6 and TNF‐αin PBS‐control, Cefalexin rabbits and LL‐37‐treated rabbits at 1h, 12 h, 1 day, 2 day and 7 day following operation (mean ± SD, *, (A) compared with PBS‐control group, *P* < 0.05; (B) compared with Cefalexin group, *P* < 0.05). Serum concentrations of C‐reactive protein (CRP), IL‐6 and TNF‐α in LL‐37‐treated rabbits reduced siginificantly compared with PBS‐control and Cefalexin rabbits from 1 day.

The levels of IL‐6, TNF‐α, and CRP reached a maximum at 2 days following the operation. After the injection of LL‐37, there was an increase in the serum IL‐6, TNF‐α, and CRP contents in rabbits in both groups; however, from 1 day the level of serum IL‐6 (*P* = 0.034), TNF‐α (*P* = 0.043), and CRP (*P* = 0.039) decreased significantly compared to the Cefalexin and control group (Fig. 4). At 7 days, the level of serum IL‐6 (*P* = 0.029), TNF‐α (*P* = 0.033), and CRP (*P* = 0.027) has reverted to normal levels in LL‐37 groups.

## Discussion

### *The Antibacterial Effect of Cationic Peptide LL‐37*

Orthopaedic clinical infection presents a difficult problem^4^. Patients may suffer complications such as delayed healing and chronic osteomyelitis after infection, which often leads to failure of surgery^13^. In the middle of the 20th century, orthopaedic patients would get infected with Gram‐positive coccus bacteria, mainly *Staphylococcus aureus* and *S. pyogenes* ^14^. In the last 50 years, great changes have occurred in the type of causative pathogenic microorganisms that infect the host. Exogenous bacterial infections have been gradually reduced, while the endogenous “normal flora” or “nonpathogenic bacteria” invading from the surroundings owing to low resistance of the host have been on the rise^15^.

*S. aureus* often leads to sepsis, colonizes catheters, causes pneumonia and wound infections, and is still one of the most common pathogenic bacteria in fracture infections^16^. Studies have shown that the *S. aureus* were found in 58% of the infections after internal fixation^17^. *Staphylococcus* can exist in the internal fixation of the surface for a long time, especially in the power screw in the large surface area, which can get implanted in bone tissue and gradually breed until they give rise to clinical symptoms. Although the incidence of orthopaedic clinical infections has been significantly reduced by using antibiotics, multidrug resistant bacteria pose a significant problem^17^. Treatment of multidrug resistant *S. aureus* infections may be complicated^18^, and a potent drug to which *S. aureus* is sensitive, is necessary.

Bioglass is one of the most important inorganic biomedical materials and has been used in orthopaedic treatment as non‐antibiotic approaches^19^. Antimicrobial peptides (AMP) are a kind of peptide with antimicrobial activity. When compared with traditional antibiotics, most antimicrobial peptides have a broad spectrum and high thermal stability^20^. The mechanism of action of antimicrobial peptides has being actively studied and the majority of experiments to date have focused on the interaction of cationic peptides with model membrane systems. They are especially lethal towards drug resistant bacteria, and can selectively kill tumor cells, inhibit replication of the Hepatitis B virus, as well as perform other functions. With the increasing threat of antibiotic resistance, viral diseases, and tumors, antimicrobial peptides appear to be the ideal antibacterial, antiviral, and anticancer drugs. The application of antimicrobial peptides to solve key problems such as bacterial drug resistance appears to be very promising.

### *The Improvement of Inflammatory Cytokines*

Cytokines are small molecule polypeptide proteins produced by the body's immune or non‐immune cells, which are closely associated with hematopoietic, inflammatory, and immune reactions^21^. The cytokines IL‐6, known as inflammatory factors, could lead to inflammation and tissue damage^22^. CRP is produced by the liver cells and vascular endothelial cells upon stimulation by a variety of inflammatory factors, leading to an increase in the levels of CRP in all types of acute inflammations^23^. The level of TNF‐α is an essential cytokine for the host defense, and its depletion by treatment may facilitate the risk of infections or their reactivation. The results have showed the cationic peptide LL‐37 could siginificantly change the inflammatory cytokines levels which were consistent with previous results of similar studies^24^.

### *Limitations*

In our study, we isolated the pathogenic bacteria from only one person, however, the etiology of infection of femoral fracture after internal fixation is so complicated that the result is affected by a lot of factors on the surface of the material, and needs further research. Meanwhile, our results just showed biofilm formation and serum levels; more importantly, histological sectioning as well as radiological findings are needed. In the future, we hope to consider all aspects of different factors when conducting experiments *in vivo*.

### *Conclusion*

Cationic peptide LL‐37 could significantly reduce biofilm formation, modify biofilm composition and levels of inflammatory factors. These results indicate that LL‐37 may be one of the promising compounds in the control of femoral fracture infection after internal fixation *in vivo*.
